# Mania

**Published:** 1890-02

**Authors:** G. W. Sherbino

**Affiliations:** Abilene, Texas


### MANIA.

G. W. Sherbino, M. D., Abilene, Texas.

Mania.—Desires to bite and strike those around. Bell., Stram.

— Is afraid of imaginary things. Tries to hide himself. Bell., Arsenicum.

— Desires light and company. Stramonium.

— Desires to cut and tear everything, especially the clothes. Veratrum-alb.

— Desires to kiss everybody. Veratrum-alb.

— Desires to go home. Bry., Bell., Lach., Opium.

— Strips off his clothes. Bell., Hyos.

— Wants to go naked. Phosphorus.

— Mind wandering off at night. Bell.

— Piety, nocturnal. Stram.

— Rage. Bell., Cuprum, Hyos., Secale, Stram.

— Shame, absence of. Hyos., Phos.

— Sings amorous songs. Hyos.

— Laughs and cries. Bell.

— Sings obscene songs. Hyos., Stram.

— Talks obscene. Nux-m., Verat-alb.

— Wants to expose the sexual parts. Hyos.

— Exposes the pudenda. Hyos.

— Desires to bite and spit. Bell.

— Escape, tries to. Bell., Stram.

— Expose himself, wishes to. Hyos., Phos.

— Hide, desires to. Bell., Stram.

— Imagines that rags are fine clothing. Sulph.

— Religious. Crocus, Lach.

— With lascivious talk. Hyos., Verat-alb.

Cephalalgia.—*Pain from the frontal region, extending upward and backward to the vertex.*

*Sharp, shooting pain from left eye to the vertex.*

Bruised feeling in the brain (Baptisia), had to walk very carefully—and keep the head erect—any quick motion turning the head would aggravate the pain. Vertigo from rising from a horizontal position (Bap., Bry.). Sensation as though a large nail was driven into the right side of the vertex (see Nux-v.); this came on after a spell of chagrin.

That part of the brain that aches is from the front of the ears on one side to the other. If that much of the brain was cut out the balance would be healthy. A dull aching, aggravated by stepping from a high step to the ground. One dose of Phytolacca 45M, F., cured.

